# Two-sample Mendelian randomization study reveals no causal relationship between inflammatory bowel disease and urological cancers

**DOI:** 10.3389/fgene.2023.1275247

**Published:** 2023-12-21

**Authors:** Haoyang Zhang, Can Hu, Zhiyu Zhang, Peng Li, Gang Shen, Jiale Sun

**Affiliations:** ^1^ Department of Urology, Dushu Lake Hospital Affiliated to Soochow University, Suzhou, Jiangsu, China; ^2^ Department of Urology, Suzhou Xiangcheng People’s Hospital, Suzhou, China; ^3^ Department of Urology, The First Affiliated Hospital of Soochow University, Suzhou, Jiangsu, China

**Keywords:** inflammatory bowel disease, urological cancers, Mendelian randomization, causality, single-nucleotide polymorphisms

## Abstract

**Background:** The relationship between inflammatory bowel disease (IBD) and urological cancers has been identified in epidemiological and observational studies, while the causality remains uncertain. We examined whether IBD is causally associated with urological cancers in a Mendelian randomization (MR) study.

**Methods:** The causal relationship between IBD, its main subtypes, and urological cancers was investigated using genome-wide association study data. To obtain more reliable conclusions, all outcomes were divided into training and validation sets. Eligible single-nucleotide polymorphisms were selected as instrumental variables based on MR analysis assumptions. The inverse variance-weighted (IVW) method was employed as the main method along with four other complementary methods.

**Results:** In this two-sample MR study, no genetic evidence for the causal effect of IBD on urological cancers was found in either the training or validation sets using the IVW method. Similarly, we did not observe any significant association between Crohn’s disease or ulcerative colitis and urological cancers. The results of the other methods are in accordance with those obtained using the IVW method.

**Conclusion:** In this study, we confirmed that IBD is not a causal genetic risk factor for urological cancer in a European population.

## 1 Introduction

Inflammatory bowel disease (IBD), which includes Crohn’s disease (CD) and ulcerative colitis (UC), is a chronic inflammatory disease of the intestine that accumulates mainly in the ileum, rectum, and colon (Kappelman et al., 2007). Epidemiological studies have shown that IBD has become a global condition, and its incidence has increased, particularly in newly industrialized areas (Taleban et al., 2016; Ng et al., 2017; Mak et al., 2020). IBD is an autoimmune disease of unknown etiology characterized by chronic gastrointestinal inflammation, with an increased risk of intestinal cancer in a subset of patients (Canavan et al., 2006; Lutgens et al., 2013). Previous meta-analyses and population-based studies have established that IBD can be accompanied by extraintestinal cancers (Pedersen et al., 2010; Katsanos et al., 2011; Parisian et al., 2013; Lo et al., 2021), which are assumed to be the result of an underlying inflammatory state and immunosuppressive treatment (Axelrad et al., 2016).

Despite limited data, recent studies have shown that patients with IBD are at an increased risk of urological cancer. Population-based cohort studies have suggested that IBD, especially CD, increases the risk of kidney cancer (So et al., 2017; Feng et al., 2021a). The risk of bladder cancer is only mildly elevated in patients with CD (So et al., 2017). However, some researchers have identified potential key genes involved in CD with bladder cancer (Zheng et al., 2023). IBD is a risk factor for prostate cancer (Burns et al., 2019; Ge et al., 2020; Meyers et al., 2020), and preneoplastic changes have been observed in a mouse model of chemically induced intestinal inflammation (Desai et al., 2022). While evidence suggests a link between IBD and urological cancers, the mechanism underlying this relationship remains unclear. In addition, the association between IBD and urological cancers identified in observational studies may be biased due to confounding factors. Previous studies have not illustrated the existence of a causal relationship between IBD and urological cancers; hence, our Mendelian randomization (MR) study was conducted from a genetic perspective.

MR, an epidemiological method of analysis, employs single-nucleotide polymorphisms (SNPs) as instrumental variables (IVs) to investigate the causal relationship between exposures and outcomes, with the advantage of reducing bias due to confounding factors and reverse causality (Sekula et al., 2016; Emdin et al., 2017; Davies et al., 2018). In this two-sample MR study, we attempted to examine whether IBD is causally associated with urological cancers.

## 2 Methods

### 2.1 Study design description

**FIGURE 1:**
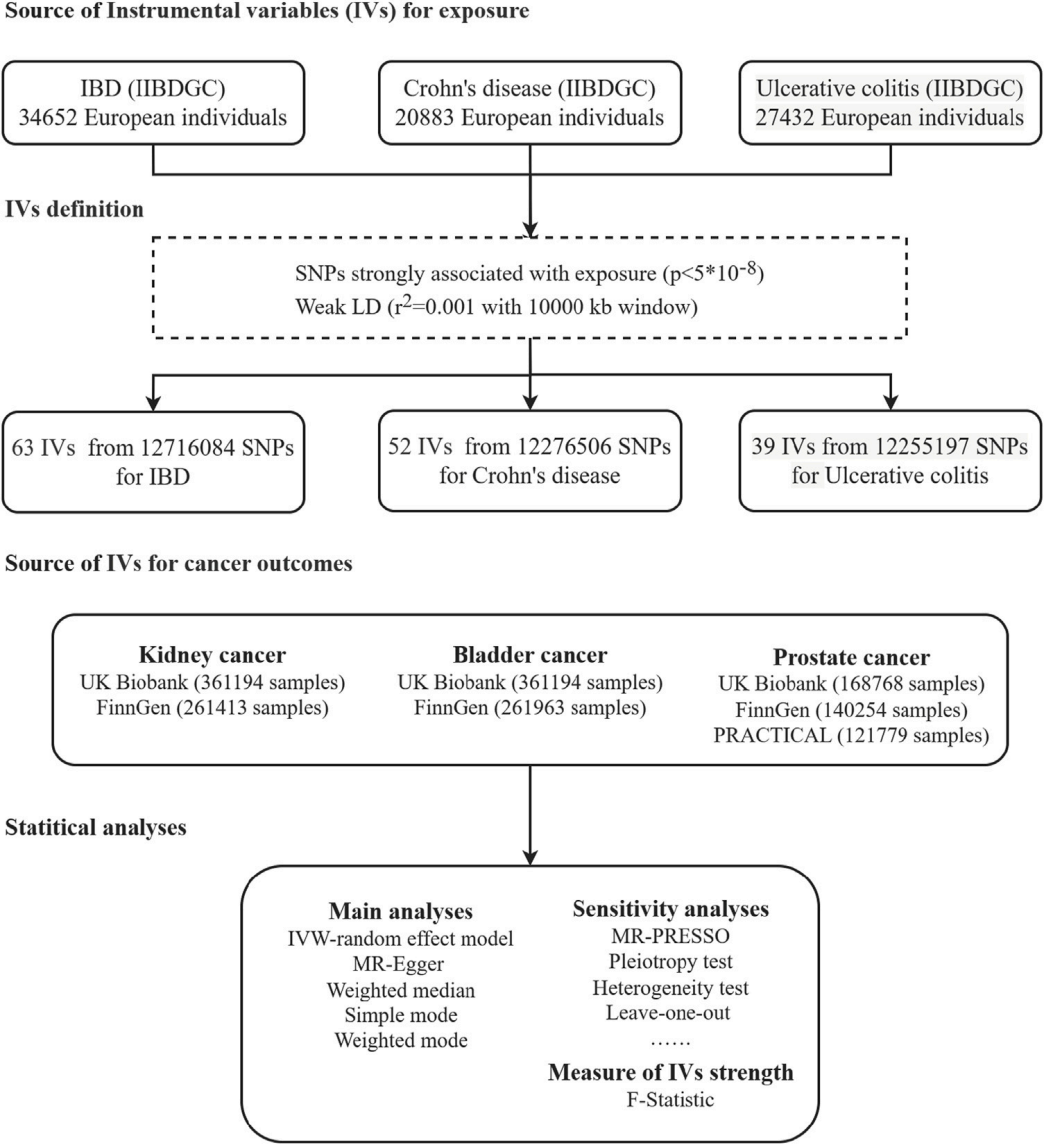
Flowchart of the Mendelian randomization analysis of Crohn’s disease and ulcerative colitis and the risk of development of kidney cancer, bladder cancer, and prostate cancer. IIBDGC, International Inflammatory Bowel Disease Genetics Consortium; IBD, inflammatory bowel disease; PRACTICAL, Prostate Cancer Association Group to Investigate Cancer-Associated Alterations in the Genome; LD, linkage disequilibrium; and SNPs, single-nucleotide polymorphisms.

Figure 1 shows our two-sample MR design comparing IBD and its main subtypes (CD and UC) with urological cancers. We split the data into training and validation sets using summary statistics from a genome-wide association study (GWAS) to investigate the causal relationship between IBD and urological cancer. Fourteen MR analyses were performed, considering IBD and its main subtypes as exposures and urological cancers as outcomes. All the participants included in this study were European. No ethical approval was required, as the data for our study were sourced from publicly available summary statistics.

### 2.2 Filtering of instrumental variables for MR analyses

The following steps were used to select instrumental variables to ensure the accuracy and authenticity of the study: first, SNPs significantly associated with urological cancers and IBD were selected at a genome-wide significance threshold (*p* < 5 × 10^–8^). Second, we conducted linkage disequilibrium (LD) analyses (r^2^ = 0.001 and with 10000 kb window) among the included instrumental variables. Third, SNPs associated with the outcome (*p* < 5 × 10^–8^) were excluded. The strength of the instrument was evaluated using the formula F = (n-k-1/k) × R^2^ × (1-R^2^), where n denotes the sample size, k denotes the number of selected instrumental variables, and R^2^ is the exposure variance explained by the included SNPs. F < 10 indicates a weak association between instrumental variables and exposures (Burgess et al., 2011).

### 2.3 Selection of data sources and instrumental variables for IBD

We considered IBD, including overall IBD, CD, and UC, as exposure. Statistics on IBD obtained from European cases were downloaded from the International IBD Genetics Consortium (IIBDGC) (Liu et al., 2015), with 12,882 cases, 21,770 controls, and 12,716,084 SNPs in IBD, 5,956 cases, 14,927 controls, and 12,276,506 SNPs in CD, and 6,968 cases, 20,464 controls, and 12,255,197 SNPs in UC (Supplementary Material; Table 1).

**TABLE 1:** Characteristics of GWAS on the exposures and outcomes.

Exposure	Consortium	Total population	Total SNPs	Cases	Controls	Ethnicity	Reference
Inflammatory bowel disease	IIBDGC	34,652	12,716,084	12,882	21,770	European	PMID 26192919
Crohn’s disease	IIBDGC	20,883	12,276,506	5,956	14,927	European	PMID 26192919
Ulcerative colitis	IIBDGC	27,432	12,255,197	6,968	20,464	European	PMID 26192919

Outcome	Consortium	Total population	Total SNPs	Cases	Controls	Ethnicity	References
Kidney cancer	UK Biobank	361,194	13,791,467	701	360,493	European	https://www.ukbiobank.ac.uk/
	FinnGen	261,413	16,380,308	1830	259,583	European	PMID 36653562
Bladder cancer	UK Biobank	361,194	13,791,467	1,554	359,640	European	https://www.ukbiobank.ac.uk/
	FinnGen	261,963	16,380,305	2,380	259,583	European	PMID 36653562
Prostate cancer	UK Biobank	168,748	13,791,467	7,872	160,876	European	https://www.ukbiobank.ac.uk/
	FinnGen	140,254	16,377,987	79,148	61,106	European	PMID 36653562
	PRACTICAL	121,779	20,346,368	11,590	110,189	European	PMID 29892016

PRACTICAL, Prostate Cancer Association Group to Investigate Cancer-Associated Alterations in the Genome; SNPs, single-nucleotide polymorphisms.

### 2.4 Selection of data sources and instrumental variables for urological cancers

We selected kidney, bladder, and prostate cancers as outcomes, and each outcome was divided into training and validation sets. Two validation sets were used for prostate cancer. All related statistics of the training sets are available in the UK Biobank (https://www.ukbiobank.ac.uk/), in which SNPs associated with kidney cancer were obtained from 701 European cases and 360,493 controls, SNPs associated with bladder cancer were obtained from 1,554 cases and 359,640 controls, and SNPs associated with prostate cancer were obtained from 7,872 cases and 160,876 controls. The datasets for the validation sets were obtained from FinnGen (Kurki et al., 2023), with 1,830 cases and 259,583 controls in kidney cancer, 2,380 cases and 259,583 controls in bladder cancer, and 79,148 cases and 61,106 controls in prostate cancer. The data for the other validation set in prostate cancer were obtained from the Prostate Cancer Association Group to Investigate Cancer-Associated Alterations in the Genome (PRACTICAL) consortium (11,590 cases and 110,189 controls) (Schumacher et al., 2018). Detailed data are shown in Table 1.

### 2.5 Statistical analysis

Five different methods were used, namely, inverse variance-weighted (IVW), MR-Egger, weighted median, simple mode, and weighted mode. IVW was the main analysis used for assessing whether there was a causal effect of IBD on urological cancers. IVW can evaluate the causal effects of genetically predicted exposure on outcomes using a weighted regression of SNP-specific Wald ratios when no horizontal pleiotropy exists. We performed MR-Egger regression analysis for assessing whether there was potential horizontal pleiotropy in the included SNPs. Potential outliers reflecting likely pleiotropic biases were detected by performing Mendelian randomization pleiotropy residual sum and outlier (MR-PRESSO), and any outlying SNP was excluded to correct for horizontal pleiotropic effects. Cochran’s Q statistic was used to examine the heterogeneity among the selected SNPs. We also applied a leave-one-out sensitivity analysis to identify whether there was a potentially strong influence of SNPs. The results of the MR analyses are expressed as odds ratios (ORs) and 95% confidence intervals (CIs) to estimate the degree of the causal effect. All analyses were conducted using the TwoSampleMR package (version 0.5.6) in R software (version 4.2.3, https://www.r-project.org/).

## 3 Results

### 3.1 Selected genetic instrumental variables

We screened IVs according to the criteria described above and selected 63, 52, and 39 independent SNPs as IVs for IBD, CD, and UC, respectively. The F-statistics revealed no weak instrumental bias between the screened IVs and exposure (all F > 10). Detailed information on the selected SNPs and F analyses is presented in Supplementary Tables S1–S3. The IVs of exposure used in 14 MR analyses (IBD and urological cancers) shared the same filtered SNPs.

### 3.2 Causal effect of IBD on kidney cancer

**FIGURE 2:**
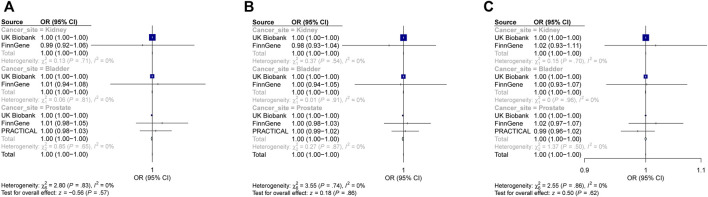
Forest plot: causal associations of inflammatory bowel disease and its main subtypes with urological cancers using the IVW method. **(A)** Effect of inflammatory bowel disease on urological cancers. **(B)** Effect of Crohn’s disease on urological cancers. **(C)** Effect of ulcerative colitis on urological cancers. PRACTICAL, Prostate Cancer Association Group to Investigate Cancer-Associated Alterations in the Genome; IVW, inverse variance-weighted; OR, odds ratio; and CI, confidence interval.

IVW results showed that overall IBD, CD, and UC were not causally related to kidney cancer in either the training set (overall IBD: OR = 1.000002, 95%CI = 0.9998–1.0002, *p* = 0.986; CD: OR = 1.00007, 95%CI = 0.9999–1.0002, *p* = 0.468; and UC: OR = 1.0001, 95%CI = 0.9999–1.0004, *p* = 0.343) or the validation set (overall IBD: OR = 0.987, 95%CI = 0.918–1.061, *p* = 0.714; CD: OR = 0.982, 95%CI = 0.927–1.041, *p* = 0.546; and UC: OR = 1.018, 95%CI = 0.932–1.111, *p* = 0.699). The overall effect of the training and validation sets also indicated no significant association between IBD and kidney cancer, as shown in the forest plot drawn based on the IVW method (Figure 2). We also drew a forest plot based on the MR-Egger method, and the results were consistent (Supplementary Figure S1). Scatter and forest plots derived from the IVW method are shown in Supplementary Figures S2, S3. No significant associations were observed in the other four models as well. Detailed information on MR analyses is presented in Supplementary Tables S4–S6. Therefore, we believe that IBD and its main subtypes are not causal risk factors for kidney cancer in European populations.

### 3.3 Causal effect of IBD on bladder cancer

Similarly, overall IBD, CD, and UC were not significantly associated with bladder cancer using the IVW method in the training set (overall IBD: OR0.9999, 95%CI = 0.9995–1.0002, *p* = 0.482; CD: OR = 0.99995, 95%CI = 0.9997–1.0002, *p* = 0.704; and UC: OR = 0.9999, 95%CI = 0.9995–1.0003, *p* = 0.556). Data from the validation set showed similar results (overall IBD: OR = 1.008, 95%CI = 0.943–1.077, *p* = 0.817); CD: OR = 0.997, 95%CI = 0.941–1.055, *p* = 0.906; and UC: OR = 1.002, 95%CI = 0.935–1.074, *p* = 0.958). Forest plots depicting the overall effect also indicated that IBD and its major subtypes were not associated with bladder cancer based on the IVW (Figure 2) and MR-Egger methods (Supplementary Figure S1). Supplementary Figures S2, S3 show the scatter and forest plots based on the IVW method. No correlation was observed with the other methods (Supplementary Tables S4–S6). Hence, there was no causal effect of IBD, CD, or UC on bladder cancer in the European population.

### 3.4 Causal effect of IBD on prostate cancer

Although observational studies suggested a correlation between IBD and prostate cancer (Burns et al., 2019; Ge et al., 2020; Meyers et al., 2020), the application of the IVW method in the training set indicates that neither the overall IBD nor CD and UC had causal effects on prostate cancer (overall IBD: OR0.9992, 95%CI = 0.998–1.0004, *p* = 0.192; CD: OR = 0.9992, 95%CI = 0.998–1.0002, *p* = 0.125; and UC: OR = 0.9999, 95%CI = 0.9984–1.001, *p* = 0.882). The data from validation sets including FinnGen (overall IBD: OR = 1.014, 95%CI = 0.998–1.051, *p* = 0.458; CD: OR = 1.005, 95%CI = 0.976–1.034, *p* = 0.741; and UC: OR = 1.018, 95%CI = 0.971–1.067, *p* = 0.459) and PRACTICAL consortium (overall IBD: OR = 1.005, 95%CI = 0.983–1.027, *p* = 0.667; CD: OR = 1.002, 95%CI = 0.986–1.019, *p* = 0.795; and UC: OR = 0.987, 95%CI = 0.958–1.016, *p* = 0.361) also indicated no significant association. Even when the overall effects of the training and validation sets were analyzed, the results did not show a noticeable tendency to be relevant depending on the IVW method (Figure 2), which was also uniform under the MR-Egger method. We also sketched the results using the IVW method in scatter and forest plots (Supplementary Figures S2, S3). Although we observed a slight causal effect of UC on prostate cancer in the PRACTICAL database only under the weighted-mode method (validation set, OR = 0.956, 95%CI = 0.917–0.998, *p* = 0.043), the genetic association was not significant in any of the other groups (Supplementary Tables S4–S6). Thus, we concluded that IBD and its main subtypes cannot serve as genetically predicted causal risk factors for prostate cancer in the European population.

### 3.5 Sensitivity analysis

Pleiotropy and heterogeneity analyses were performed to validate the reliability of MR analyses (Table 2). No incidence of potential pleiotropy was identified in the MR-Egger regression analyses or MR-PRESSO tests. Outlier SNPs were excluded using the MR-PRESSO method. Additionally, we applied Cochran’s Q *p*-value (Table 2) and funnel plots (Supplementary Figure S4) to IVW and MR-Egger methods, and heterogeneity was found in some analyses; such heterogeneity was acceptable in the MR study. The results of the leave-one-sensitivity analyses demonstrated that the estimates of the causal effects of the genetic prediction of IBD on urological cancers were reliable (Supplementary Figure S5).

**TABLE 2:** Pleiotropy and heterogeneity analyses.

Exposure	Outcome	Consortium	No. of SNPs	MR-Egger regression		MR-PRESSO	Heterogeneity analyses		
				Intercept	p_intercept	Global test p	Method	Q	Q_pval
Inflammatory bowel disease	Kidney cancer	UK Biobank	54	−0.00002	0.690	0.246	MR-Egger	59.836	0.213
							IVW	60.021	0.236
		FinnGen	48	−0.03584	0.054	0.353	MR-Egger	44.926	0.517
							IVW	49.725	0.365
	Bladder cancer	UK Biobank	54	−0.00001	0.936	0.060	MR-Egger	70.191	**0.047**
							IVW	70.200	0.057
		FinnGen	48	0.00009	0.995	0.270	MR-Egger	52.834	0.227
							IVW	52.834	0.259
	Prostate cancer	UK Biobank	54	0.00018	0.469	0.399	MR-Egger	54.788	0.369
							IVW	55.349	0.386
		FinnGen	48	0.01325	0.118	0.083	MR-Egger	58.640	0.100
							IVW	61.873	0.072
		PRACTICAL	51	0.00857	0.086	0.570	MR-Egger	85.137	**0.001**
							IVW	90.458	**<0.001**
Crohn’s disease	Kidney cancer	UK Biobank	44	−0.00002	0.665	0.513	MR-Egger	42.093	0.467
							IVW	42.284	0.502
		FinnGen	42	−0.02374	0.123	0.464	MR-Egger	38.094	0.556
							IVW	40.574	0.489
	Bladder cancer	UK Biobank	44	−0.00006	0.403	0.415	MR-Egger	44.105	0.383
							IVW	44.854	0.394
		FinnGen	42	−0.01461	0.329	0.196	MR-Egger	48.049	0.179
							IVW	49.224	0.177
	Prostate cancer	UK Biobank	44	0.00015	0.543	0.142	MR-Egger	53.131	0.117
							IVW	53.608	0.129
		FinnGen	42	0.00636	0.402	0.169	MR-Egger	48.923	0.157
							IVW	49.801	0.163
		PRACTICAL	41	0.00176	0.675	0.548	MR-Egger	59.553	**0.019**
							IVW	59.826	**0.023**
Ulcerative colitis	Kidney cancer	UK Biobank	31	−0.00007	0.254	0.342	MR-Egger	31.812	0.328
							IVW	33.296	0.310
		FinnGen	29	−0.03471	0.219	0.153	MR-Egger	33.895	0.169
							IVW	35.880	0.146
	Bladder cancer	UK Biobank	31	−0.00010	0.364	0.057	MR-Egger	42.888	**0.047**
							IVW	44.147	**0.046**
		FinnGen	29	−0.00439	0.844	0.873	MR-Egger	19.788	0.840
							IVW	19.827	0.871
	Prostate cancer	UK Biobank	31	0.00050	0.222	0.550	MR-Egger	48.632	**0.013**
							IVW	51.240	**0.009**
		FinnGen	28	−0.00652	0.666	0.154	MR-Egger	46.860	**0.007**
							IVW	47.204	**0.009**
		PRACTICAL	29	0.00364	0.654	0.137	MR-Egger	76.451	**<0.001**
							IVW	77.033	**<0.001**

PRACTICAL, Prostate Cancer Association Group to Investigate Cancer-Associated Alterations in the Genome; IVW, inverse variance-weighted; MR-PRESSO, Mendelian randomization pleiotropy residual sum and outlier; SNPs, single‐nucleotide polymorphisms. Bold values indicate significant difference. (*p* < 0.05).

## 4 Discussion

We appraised the genetic overlap and potential causal relationships between IBD and urological cancers using GWAS summary statistics; however, no genetic evidence for the causal effect of IBD on urological cancers was found in this two-sample MR study. The results of the validation set were consistent. We confirmed that IBD was not a causal genetic risk factor for urological cancers in the European population.

Although we did not obtain significant results, several hypotheses have been proposed regarding the associations observed in previous observational and epidemiological studies. First, the relationship between IBD and the increased risk of urological cancers remains controversial. Given scarce studies (Feng et al., 2021a; Feng et al., 2021b), speculations regarding the association between IBD and kidney cancer await confirmation. In the present meta-analysis, patients with IBD showed a trend toward an increased risk of bladder cancer in the CD subgroup (Geng and Geng, 2021). However, the association between IBD and bladder cancer was not observed to be statistically significant in another study (Feng et al., 2021b). IBD was not associated with an increased risk of prostate cancer in this population-based retrospective cohort study (Na et al., 2022). In another meta-analysis, the increased risk of prostate cancer compared with that in the general population was only little (Carli et al., 2020). Second, IBD and its main subtypes have been demonstrated to increase the risk of extraintestinal malignancies, assumed to be a result of an underlying inflammatory state and immunosuppressive therapies (Axelrad et al., 2016). Inflammation is assumed to play a role in the well-established association between IBD and the development of various solid tumors. Chronic kidney disease (CKD) is a risk factor for kidney cancer (Stengel, 2010). The potential mechanism connecting CKD and IBD involves immunoglobulin A (IgA) nephropathy, which is believed to be related to mucosal inflammation (Ambruzs et al., 2014). Patients with IBD probably have an increased risk of kidney cancer through the IBD–CKD pathway. Inflammation may lead to the activation of angiogenesis, which ultimately leads to bladder cancer progression in patients with IBD (Wigner et al., 2021). Chronic inflammation can cause DNA damage and promote carcinogenic epigenetic alterations, which may contribute to prostate tumorigenesis (Sfanos et al., 2018). In addition, a range of drugs used for the treatment of IBD, such as immunomodulators and biological agents, may increase the risk of extraintestinal cancer (Mason and Siegel, 2013). A population-based cohort study also concluded that patients with IBD who were chronically exposed to immunosuppressive treatment may be at a higher risk of being diagnosed with cancer (Van Den Heuvel et al., 2016). Patients with IBD receiving thiopurines are more likely to develop urinary tract cancers, including kidney and bladder cancer (Bourrier et al., 2016). Previous observational studies may not eliminate bias in the risk of cancer due to the use of immunosuppressive medications in patients with IBD. However, no direct evidence has been provided to accurately elucidate the correlation between immunosuppressive drugs and the risk of prostate cancer in patients with IBD. Moreover, patients with chronic inflammatory diseases commonly have more frequent healthcare utilization, which could be explained by a detection bias (Beckmann et al., 2019). Due to the nature of the disease, men with IBD have a higher prevalence of rectal examinations. Cancer can be detected during routine medical tests, with an increasing number of physician office visits over time.

The application of comprehensive GWAS data for MR analyses improves the precision of the estimated effects. The most significant advantage of this study is that causal estimates were obtained from MR, avoiding reverse causality and confounding bias. To the best of our knowledge, this is the first MR analysis on this subject. However, our MR study had some limitations. First, the mechanisms of cancers caused by IBD are complex, and this study only offers genetic evidence for the absence of a causal relationship between IBDs and urological cancers. Therefore, they cannot provide detailed temporal information on disease development. Integrating MR results with long-term clinical observations and patient data may contribute to a more comprehensive understanding. Second, some heterogeneity could not be avoided using Cochran’s Q *p*-value in the previous MR analyses. In addition, MR studies are limited to specific races; thus, conclusions drawn from the analyses of populations of different races are invalid. Therefore, our results are limited to the European population. Finally, the MR method relies on certain assumptions, and there may be instances of improper IV selection or failure that account for potential confounding factors.

In conclusion, our MR study demonstrated that IBD has no causal effect on urological cancers in either the training or validation sets in European populations. Considering that patients with IBD are at an increased risk of developing cancer, further studies are needed to explore the relationship between IBD and cancer.

## Data Availability

The original contributions presented in the study are included in the article/Supplementary Material; further inquiries can be directed to the corresponding authors.

## Supplementary material

SUPPLEMENTARY FIGURE S1 Forest plot: Causal associations of inflammatory bowel disease and its main subtypes with urological cancers using the MR‐Egger method. **(A)** Effect of inflammatory bowel disease on urological cancers. **(B)** Effect of Crohn's disease on urological cancers. **(C)** Effect of ulcerative colitis on urological cancers. PRACTICAL, Prostate Cancer Association Group to Investigate Cancer-Associated Alterations in the Genome; OR, odds ratio; and CI, confidence interval.

SUPPLEMENTARY FIGURE S2 Scatter plots of the association between inflammatory bowel disease and its main subtypes and urological cancers. The five methods applied in this study were all demonstrated. Lines in different colors represent IVW, MR-Egger, weighted median, simple mode, and weight mode methods. PRACTICAL, Prostate Cancer Association Group to Investigate Cancer-Associated Alterations in the Genome; IVW, inverse variance-weighted; and SNP, single-nucleotide polymorphism.

SUPPLEMENTARY FIGURE S3 Funnel plots were applied to detect whether the observed association was along with obvious heterogeneity using IVW and MR-Egger methods. PRACTICAL, Prostate Cancer Association Group to Investigate Cancer-Associated Alterations in the Genome; IVW, inverse variance-weighted.

SUPPLEMENTARY FIGURE S4 Leave-one-out analyses were applied to evaluate whether any single instrumental variable was driving the causal effect. PRACTICAL, Prostate Cancer Association Group to Investigate Cancer-Associated Alterations in the Genome.

SUPPLEMENTARY FIGURE S5 Forest plots were applied to demonstrate the MR estimate and 95%CI values for each SNP, which also show the MR-Egger and IVW results at the bottom. PRACTICAL, Prostate Cancer Association Group to Investigate Cancer-Associated Alterations in the Genome; IVW, inverse variance-weighted.

## References

[B1] Ambruzs J. M. Walker P. D. Larsen C. P. (2014). The histopathologic spectrum of kidney biopsies in patients with inflammatory bowel disease. Clin. J. Am. Soc. Nephrol. 9 (2), 265–270. 10.2215/CJN.04660513 24262508 PMC3913236

[B2] Axelrad J. E. Lichtiger S. Yajnik V. (2016). Inflammatory bowel disease and cancer: the role of inflammation, immunosuppression, and cancer treatment. World J. Gastroenterol. 22 (20), 4794–4801. 10.3748/wjg.v22.i20.4794 27239106 PMC4873872

[B3] Beckmann K. Russell B. Josephs D. Garmo H. Haggstrom C. Holmberg L. (2019). Chronic inflammatory diseases, anti-inflammatory medications and risk of prostate cancer: a population-based case-control study. BMC Cancer 19 (1), 612. 10.1186/s12885-019-5846-3 31226970 PMC6588859

[B4] Bourrier A. Carrat F. Colombel J. F. Bouvier A. M. Abitbol V. Marteau P. (2016). Excess risk of urinary tract cancers in patients receiving thiopurines for inflammatory bowel disease: a prospective observational cohort study. Aliment. Pharmacol. Ther. 43 (2), 252–261. 10.1111/apt.13466 26549003

[B5] Burgess S. Thompson S. G. CRP CHD Genetics Collaboration (2011). Avoiding bias from weak instruments in Mendelian randomization studies. Int. J. Epidemiol. 40 (3), 755–764. 10.1093/ije/dyr036 21414999

[B6] Burns J. A. Weiner A. B. Catalona W. J. Schaeffer E. M. Hanauer S. B. (2019). Inflammatory bowel disease and the risk of prostate cancer. Eur. Urol. 75 (5), 846–852. 10.1016/j.eururo.2018.11.039 30528221 PMC6542355

[B7] Canavan C. Abrams K. R. Mayberry J. (2006). Meta-analysis: colorectal and small bowel cancer risk in patients with Crohn's disease. Aliment. Pharmacol. Ther. 23 (8), 1097–1104. 10.1111/j.1365-2036.2006.02854.x 16611269

[B8] Carli E. Caviglia G. P. Pellicano R. Fagoonee S. Rizza S. Astegiano M. (2020). Incidence of prostate cancer in inflammatory bowel disease: a meta-analysis. Med. Kaunas. 56 (6), 285. 10.3390/medicina56060285 PMC7353864 32545154

[B9] Davies N. M. Holmes M. V. Davey Smith G. (2018). Reading Mendelian randomisation studies: a guide, glossary, and checklist for clinicians. Bmj 362, k601. 10.1136/bmj.k601 30002074 PMC6041728

[B10] Desai A. S. Sagar V. Lysy B. Weiner A. B. Ko O. S. Driscoll C. (2022). Inflammatory bowel disease induces inflammatory and pre-neoplastic changes in the prostate. Prostate Cancer Prostatic Dis. 25 (3), 463–471. 10.1038/s41391-021-00392-7 34035460 PMC8647933

[B11] Emdin C. A. Khera A. V. Kathiresan S. (2017). Mendelian randomization. Jama 318 (19), 1925–1926. 10.1001/jama.2017.17219 29164242

[B12] Feng D. Bai Y. Liu S. Yang Y. Han P. Wei W. (2021a). Risk of renal cancer in patients with inflammatory bowel disease: a pooled analysis of population-based studies. Urol. Oncol. 39 (2), 93–99. 10.1016/j.urolonc.2020.10.078 33214029

[B13] Feng D. Yang Y. Wang Z. Wei W. Li L. (2021b). Inflammatory bowel disease and risk of urinary cancers: a systematic review and pooled analysis of population-based studies. Transl. Androl. Urol. 10 (3), 1332–1341. 10.21037/tau-20-1358 33850767 PMC8039624

[B14] Ge Y. Shi Q. Yao W. Cheng Y. Ma G. (2020). The association between inflammatory bowel disease and prostate cancer risk: a meta-analysis. Prostate Cancer Prostatic Dis. 23 (1), 53–58. 10.1038/s41391-019-0177-7 31591455

[B15] Geng Z. Geng Q. (2021). Risk of urinary bladder cancer in patients with inflammatory bowel diseases: a meta-analysis. Front. Surg. 8, 636791. 10.3389/fsurg.2021.636791 34124132 PMC8188732

[B16] Kappelman M. D. Rifas-Shiman S. L. Kleinman K. Ollendorf D. Bousvaros A. Grand R. J. (2007). The prevalence and geographic distribution of Crohn's disease and ulcerative colitis in the United States. Clin. Gastroenterol. Hepatol. 5 (12), 1424–1429. 10.1016/j.cgh.2007.07.012 17904915

[B17] Katsanos K. H. Tatsioni A. Pedersen N. Shuhaibar M. Ramirez V. H. Politi P. (2011). Cancer in inflammatory bowel disease 15 years after diagnosis in a population-based European Collaborative follow-up study. J. Crohns Colitis 5 (5), 430–442. 10.1016/j.crohns.2011.04.013 21939917

[B18] Kurki M. I. Karjalainen J. Palta P. Sipilä T. P. Kristiansson K. Donner K. M. (2023). FinnGen provides genetic insights from a well-phenotyped isolated population. Nature 613 (7944), 508–518. 10.1038/s41586-022-05473-8 36653562 PMC9849126

[B19] Liu J. Z. Van Sommeren S. Huang H. Ng S. C. Alberts R. Takahashi A. (2015). Association analyses identify 38 susceptibility loci for inflammatory bowel disease and highlight shared genetic risk across populations. Nat. Genet. 47 (9), 979–986. 10.1038/ng.3359 26192919 PMC4881818

[B20] Lo B. Zhao M. Vind I. Burisch J. (2021). The risk of extraintestinal cancer in inflammatory bowel disease: a systematic review and meta-analysis of population-based cohort studies. Clin. Gastroenterol. Hepatol. 19 (6), 1117–1138.e19. 10.1016/j.cgh.2020.08.015 32801010

[B21] Lutgens M. W. Van Oijen M. G. Van Der Heijden G. J. Vleggaar F. P. Siersema P. D. Oldenburg B. (2013). Declining risk of colorectal cancer in inflammatory bowel disease: an updated meta-analysis of population-based cohort studies. Inflamm. Bowel Dis. 19 (4), 789–799. 10.1097/MIB.0b013e31828029c0 23448792

[B22] Mak W. Y. Zhao M. Ng S. C. Burisch J. (2020). The epidemiology of inflammatory bowel disease: east meets west. J. Gastroenterol. Hepatol. 35 (3), 380–389. 10.1111/jgh.14872 31596960

[B23] Mason M. Siegel C. A. (2013). Do inflammatory bowel disease therapies cause cancer? Inflamm. Bowel Dis. 19 (6), 1306–1321. 10.1097/MIB.0b013e3182807618 23470503

[B24] Meyers T. J. Weiner A. B. Graff R. E. Desai A. S. Cooley L. F. Catalona W. J. (2020). Association between inflammatory bowel disease and prostate cancer: a large-scale, prospective, population-based study. Int. J. Cancer 147 (10), 2735–2742. 10.1002/ijc.33048 32399975 PMC7577830

[B25] Na J. E. Kim T. J. Lee Y. C. Hong S. N. (2022). Risk of prostate cancer in patients with inflammatory bowel disease: a nationwide cohort study in South Korea. Ther. Adv. Gastroenterol. 15, 17562848221137430. 10.1177/17562848221137430 PMC9706079 36458049

[B26] Ng S. C. Shi H. Y. Hamidi N. Underwood F. E. Tang W. Benchimol E. I. (2017). Worldwide incidence and prevalence of inflammatory bowel disease in the 21st century: a systematic review of population-based studies. Lancet 390 (10114), 2769–2778. 10.1016/S0140-6736(17)32448-0 29050646

[B27] Parisian K. R. Lopez R. Shen B. (2013). Chronic pouch inflammation and risk for new-onset extraintestinal cancers in patients with restorative proctocolectomy for ulcerative colitis. Inflamm. Bowel Dis. 19 (4), 806–811. 10.1097/MIB.0b013e31827feba5 23429461

[B28] Pedersen N. Duricova D. Elkjaer M. Gamborg M. Munkholm P. Jess T. (2010). Risk of extra-intestinal cancer in inflammatory bowel disease: meta-analysis of population-based cohort studies. Am. J. Gastroenterol. 105 (7), 1480–1487. 10.1038/ajg.2009.760 20332773

[B29] Schumacher F. R. Al Olama A. A. Berndt S. I. Benlloch S. Ahmed M. Saunders E. J. (2018). Association analyses of more than 140,000 men identify 63 new prostate cancer susceptibility loci. Nat. Genet. 50 (7), 928–936. 10.1038/s41588-018-0142-8 29892016 PMC6568012

[B30] Sekula P. Del Greco M. F. Pattaro C. Köttgen A. (2016). Mendelian randomization as an approach to assess causality using observational data. J. Am. Soc. Nephrol. 27 (11), 3253–3265. 10.1681/ASN.2016010098 27486138 PMC5084898

[B31] Sfanos K. S. Yegnasubramanian S. Nelson W. G. De Marzo A. M. (2018). The inflammatory microenvironment and microbiome in prostate cancer development. Nat. Rev. Urol. 15 (1), 11–24. 10.1038/nrurol.2017.167 29089606

[B32] So J. Tang W. Leung W. K. Li M. Lo F. H. Wong M. T. L. (2017). Cancer risk in 2621 Chinese patients with inflammatory bowel disease: a population-based cohort study. Inflamm. Bowel Dis. 23 (11), 2061–2068. 10.1097/MIB.0000000000001240 28991855

[B33] Stengel B. (2010). Chronic kidney disease and cancer: a troubling connection. J. Nephrol. 23 (3), 253–262. 20349418 PMC4823382

[B34] Taleban S. Elquza E. Gower-Rousseau C. Peyrin-Biroulet L. (2016). Cancer and inflammatory bowel disease in the elderly. Dig. Liver Dis. 48 (10), 1105–1111. 10.1016/j.dld.2016.05.006 27289334

[B35] Van Den Heuvel T. R. Wintjens D. S. Jeuring S. F. Wassink M. H. H. Romberg-Camps M. J. L. Oostenbrug L. E. (2016). Inflammatory bowel disease, cancer and medication: cancer risk in the Dutch population-based IBDSL cohort. Int. J. Cancer 139 (6), 1270–1280. 10.1002/ijc.30183 27170593

[B36] Wigner P. Grębowski R. Bijak M. Saluk-Bijak J. Szemraj J. (2021). The interplay between oxidative stress, inflammation and angiogenesis in bladder cancer development. Int. J. Mol. Sci. 22 (9), 4483. 10.3390/ijms22094483 33923108 PMC8123426

[B37] Zheng Q. Guo L. Yang R. Chen Z. Liu X. (2023). Identification of essential genes and drug discovery in bladder cancer and inflammatory bowel disease via text mining and bioinformatics analysis. Curr. Comput. Aided Drug Des. 20, 359–366. 10.2174/1573409919666230330154008 37005401

