# Supplementary figures and images for: Two-sample Mendelian randomization study reveals no causal relationship between inflammatory bowel disease and urological cancers
Source: Front Genet. 2023 Dec 21;14:1275247. doi: 10.3389/fgene.2023.1275247 (PMC10771298; doi:10.3389/fgene.2023.1275247)

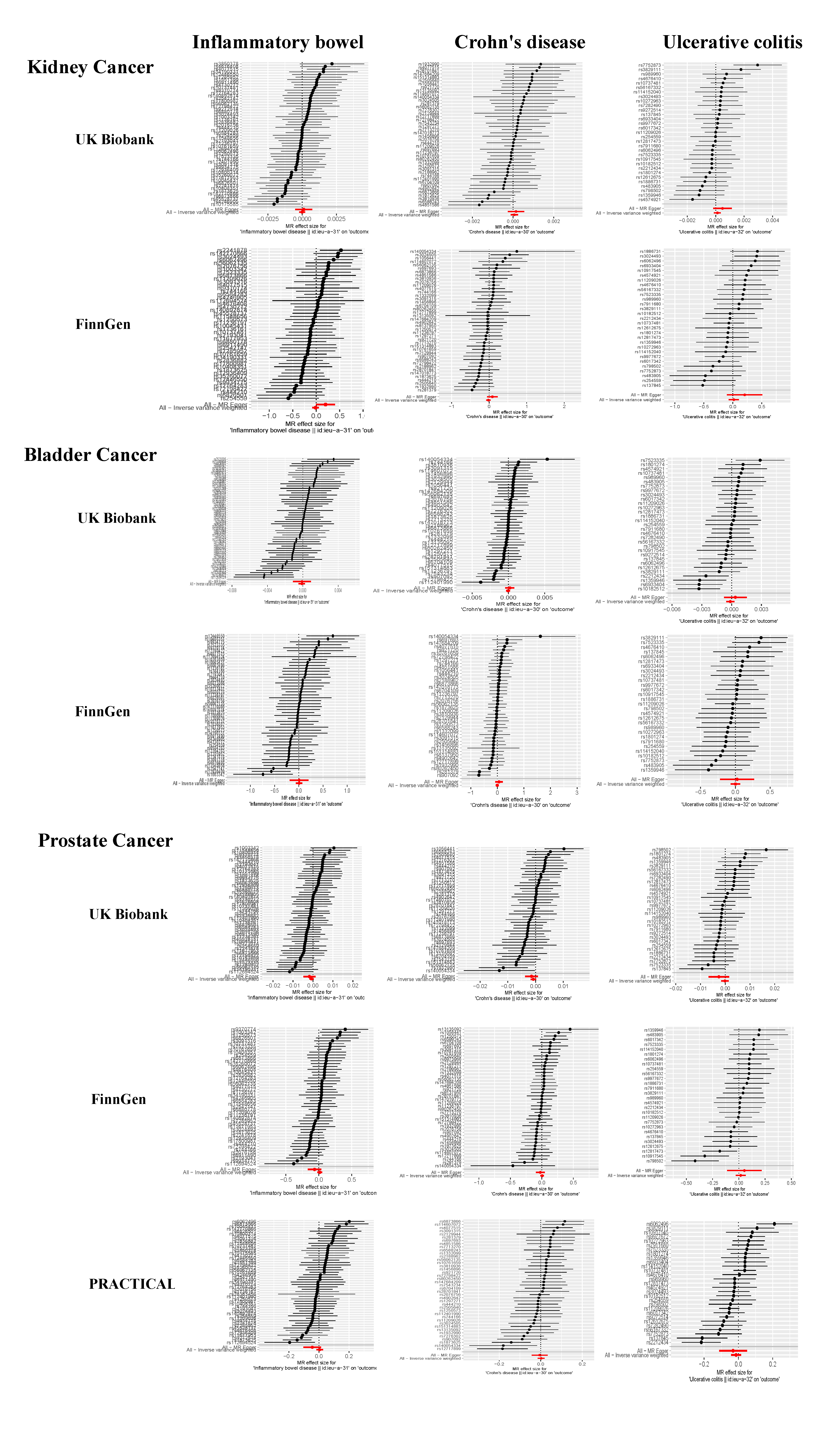

Supplement: Supplementary file 1 [file Image3.TIFF]

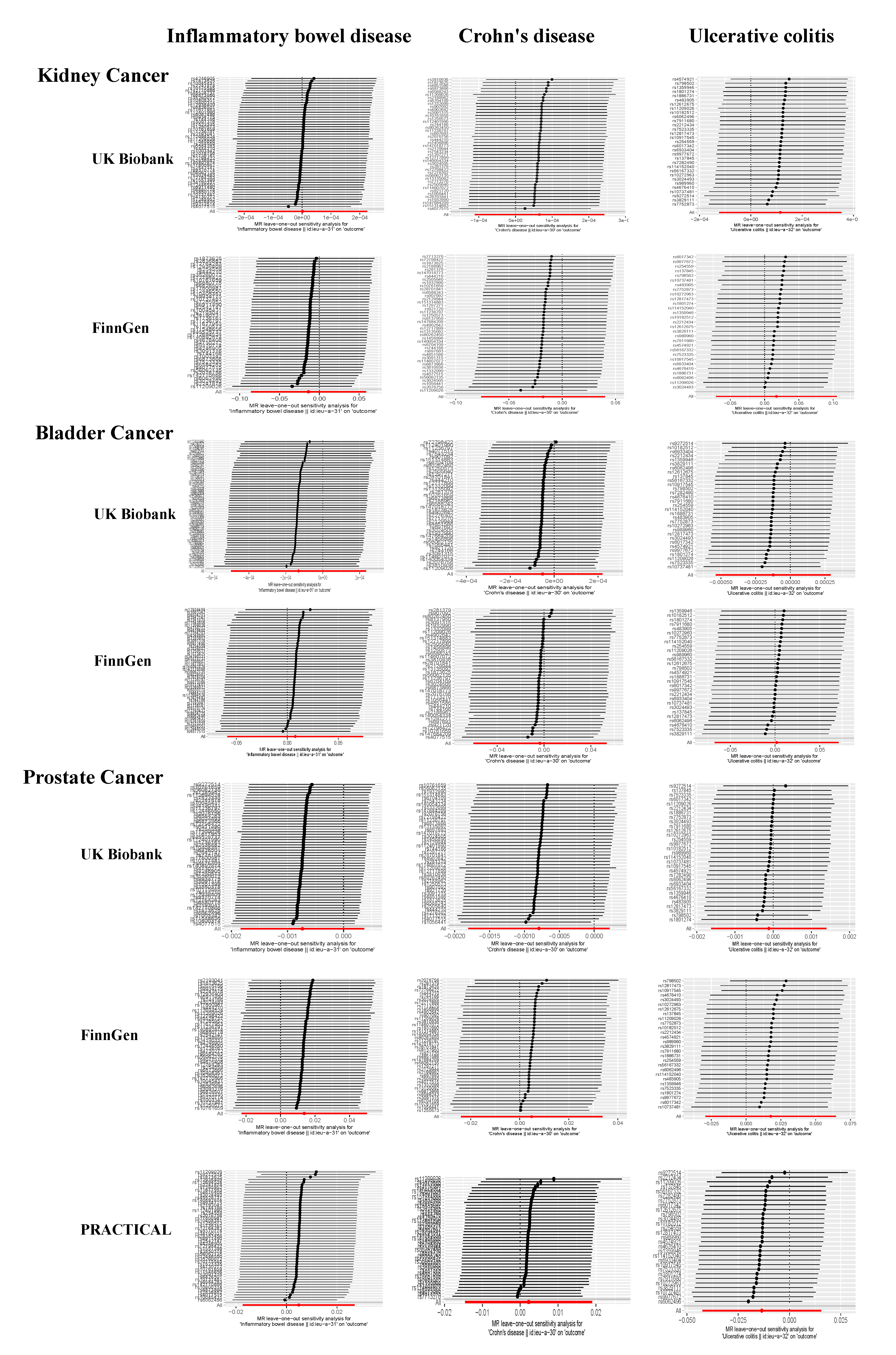

Supplement: Supplementary file 3 [file Image5.TIFF]

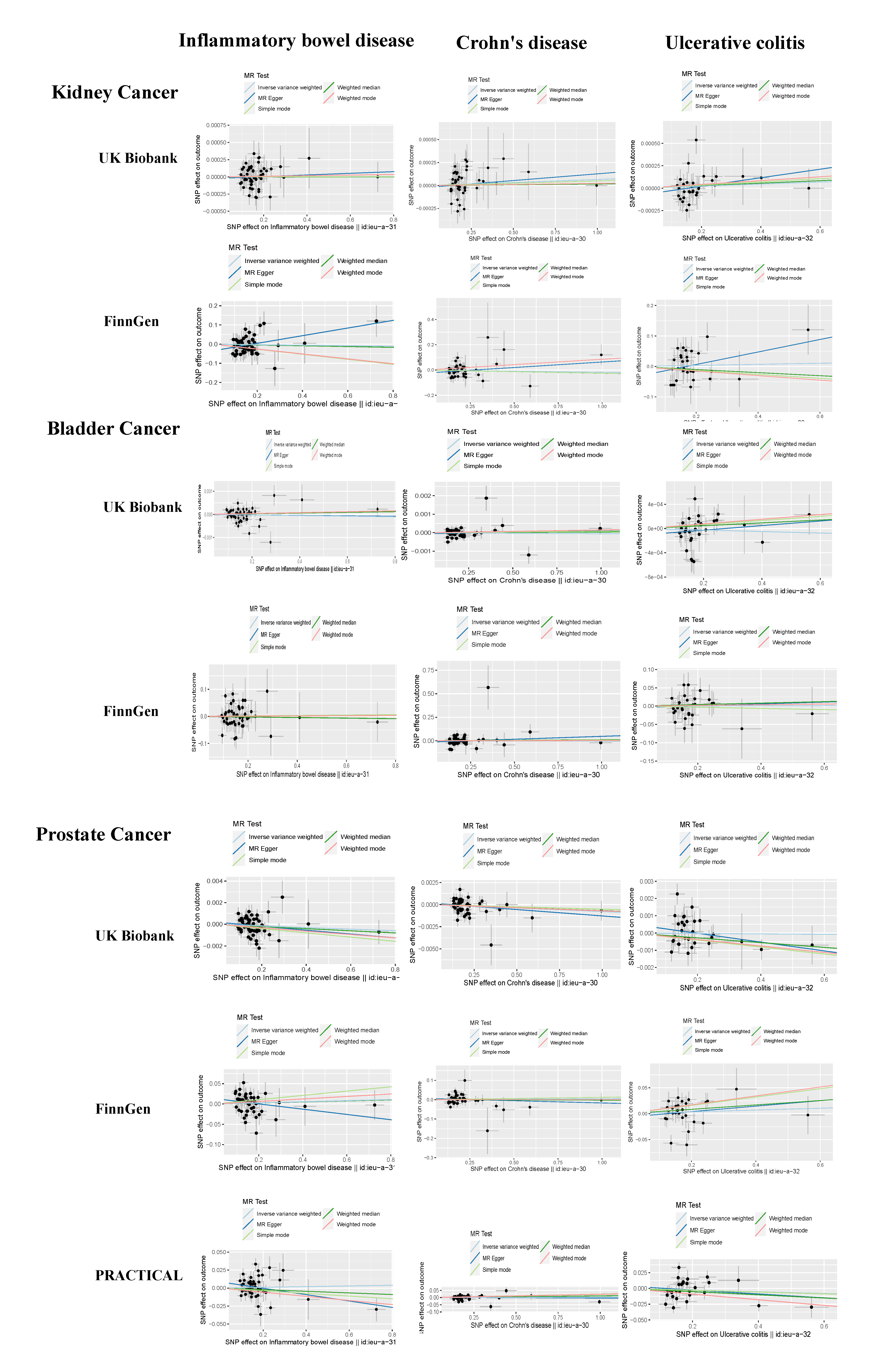

Supplement: Supplementary file 4 [file Image2.TIF]

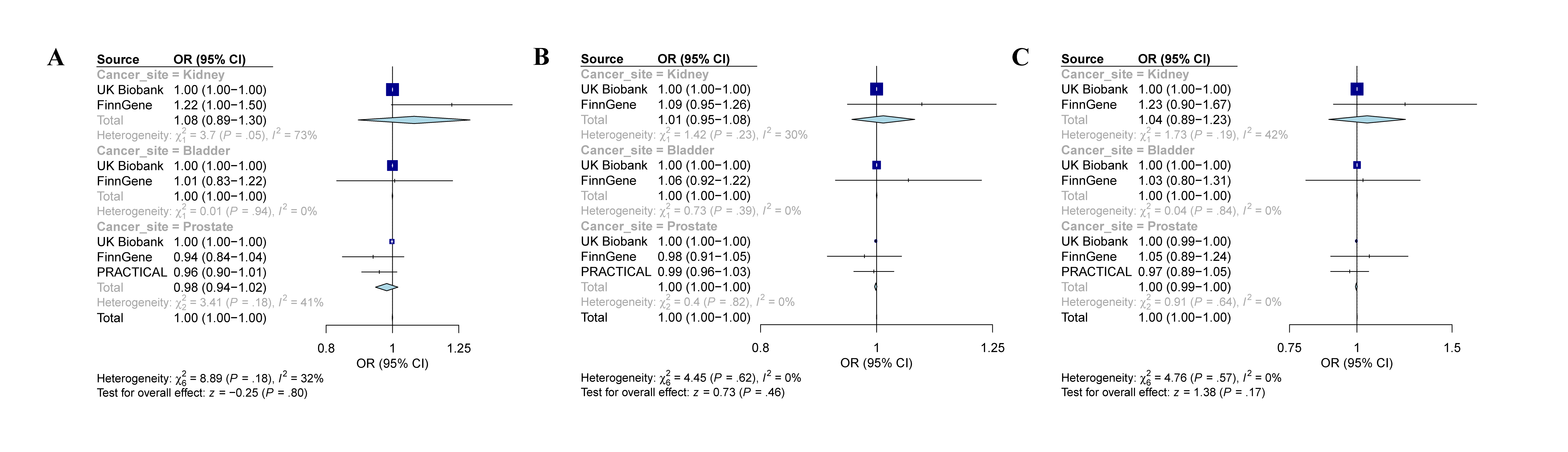

Supplement: Supplementary file 5 [file Image1.TIF]

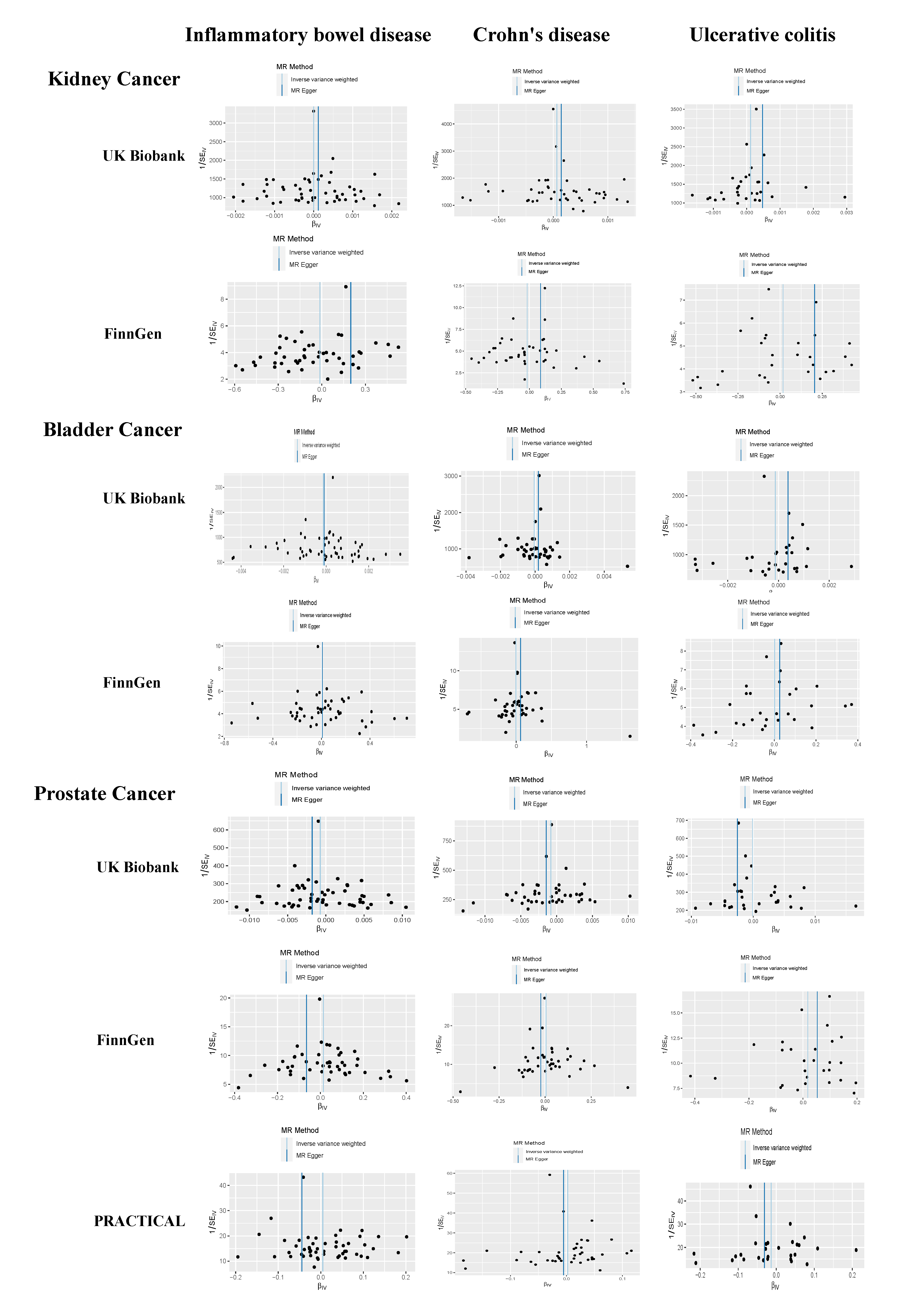

Supplement: Supplementary file 11 [file Image4.TIFF]
