# Supplementary material for: Two-sample Mendelian randomization study reveals no causal relationship between inflammatory bowel disease and urological cancers
Source: Front Genet. 2023 Dec 21;14:1275247. doi: 10.3389/fgene.2023.1275247 (PMC10771298; doi:10.3389/fgene.2023.1275247)
Supplement: Supplementary file 2 [file Table1.DOCX]

| **Table S1. F‐statistics to evaluate the instrument strength on inflammatory bowel disease.** | | | | | | | | | |
| --- | --- | --- | --- | --- | --- | --- | --- | --- | --- |
| SNP | eaf.exposure | beta.exposure | se.exposure | sample（N） | K | SD | R2 | sum(R2) | F |
| rs3024493 | 0.169666 | 0.212996 | 0.0222 | 34652 | 63 | 4.132540584 | 0.00074849 | 0.042404652 | 24.31175856 |
| rs7523335 | 0.173424 | -0.140504 | 0.0225 |  |  | 4.188385727 | 0.000322631 |  |  |
| rs10737481 | 0.554896 | 0.141103 | 0.017 |  |  | 3.164558105 | 0.000982086 |  |  |
| rs35730213 | 0.262591 | -0.151404 | 0.0194 |  |  | 3.611319249 | 0.000680709 |  |  |
| rs10800314 | 0.649219 | -0.143097 | 0.0179 |  |  | 3.332093534 | 0.000840009 |  |  |
| rs11209026 | 0.0541886 | -0.72629 | 0.0422 |  |  | 7.85555012 | 0.000876212 |  |  |
| rs112401990 | 0.375383 | 0.142202 | 0.0174 |  |  | 3.239018296 | 0.000903864 |  |  |
| rs10175585 | 0.71345 | -0.1332 | 0.0202 |  |  | 3.760239631 | 0.000513064 |  |  |
| rs4676408 | 0.515063 | 0.118103 | 0.0181 |  |  | 3.369323629 | 0.000613779 |  |  |
| rs11677953 | 0.415063 | 0.0975985 | 0.0171 |  |  | 3.183173153 | 0.000456477 |  |  |
| rs112694524 | 0.0903752 | 0.188304 | 0.0303 |  |  | 5.640359446 | 0.000183251 |  |  |
| rs2241878 | 0.538498 | 0.148001 | 0.0169 |  |  | 3.145943057 | 0.001100057 |  |  |
| rs1873625 | 0.32287 | 0.177301 | 0.0179 |  |  | 3.332093534 | 0.001237992 |  |  |
| rs6826501 | 0.536078 | -0.0927953 | 0.0169 |  |  | 3.145943057 | 0.000432766 |  |  |
| rs45528737 | 0.111833 | 0.166802 | 0.03 |  |  | 5.584514303 | 0.000177226 |  |  |
| rs254559 | 0.402063 | 0.1028 | 0.0172 |  |  | 3.2017882 | 0.000495656 |  |  |
| rs13178036 | 0.341807 | 0.100904 | 0.0184 |  |  | 3.425168772 | 0.000390496 |  |  |
| rs17800987 | 0.089179 | 0.2017 | 0.0305 |  |  | 5.677589541 | 0.000205026 |  |  |
| rs6880778 | 0.620729 | 0.187801 | 0.0173 |  |  | 3.220403248 | 0.001601242 |  |  |
| rs6873866 | 0.528821 | -0.106996 | 0.0176 |  |  | 3.276248391 | 0.000531503 |  |  |
| rs35260072 | 0.433153 | 0.142197 | 0.017 |  |  | 3.164558105 | 0.000991498 |  |  |
| rs10045431 | 0.717524 | 0.177406 | 0.0189 |  |  | 3.518244011 | 0.001030701 |  |  |
| rs148844907 | 0.0103903 | 1.1375 | 0.0963 |  |  | 17.92629091 | 8.28026E-05 |  |  |
| rs34190331 | 0.0853045 | 0.176899 | 0.0303 |  |  | 5.640359446 | 0.000153503 |  |  |
| rs28383456 | 0.345334 | -0.177501 | 0.02 |  |  | 3.723009535 | 0.001027785 |  |  |
| rs6927172 | 0.21432 | 0.110295 | 0.0202 |  |  | 3.760239631 | 0.000289747 |  |  |
| rs4712528 | 0.787692 | 0.122598 | 0.0207 |  |  | 3.853314869 | 0.000338572 |  |  |
| rs9370774 | 0.20068 | -0.130704 | 0.0219 |  |  | 4.076695441 | 0.000329774 |  |  |
| rs9272514 | 0.30636 | -0.234698 | 0.0208 |  |  | 3.871929917 | 0.001561565 |  |  |
| rs6911490 | 0.778565 | -0.142801 | 0.0208 |  |  | 3.871929917 | 0.000469006 |  |  |
| rs444210 | 0.545781 | 0.109502 | 0.0168 |  |  | 3.12732801 | 0.00060787 |  |  |
| rs4730272 | 0.512591 | -0.134102 | 0.0178 |  |  | 3.313478486 | 0.000818459 |  |  |
| rs1551399 | 0.615807 | 0.101302 | 0.0173 |  |  | 3.220403248 | 0.000468209 |  |  |
| rs4246905 | 0.729896 | 0.163001 | 0.0197 |  |  | 3.667164392 | 0.000779008 |  |  |
| rs1887428 | 0.61936 | -0.171595 | 0.0178 |  |  | 3.313478486 | 0.001264528 |  |  |
| rs4077515 | 0.424242 | 0.179401 | 0.0172 |  |  | 3.2017882 | 0.001533727 |  |  |
| rs10761659 | 0.554011 | 0.161896 | 0.0172 |  |  | 3.2017882 | 0.001263454 |  |  |
| rs12764283 | 0.337409 | 0.126597 | 0.0179 |  |  | 3.332093534 | 0.000645423 |  |  |
| rs1250573 | 0.295706 | -0.113606 | 0.019 |  |  | 3.536859059 | 0.000429745 |  |  |
| rs6584283 | 0.510643 | -0.180303 | 0.0169 |  |  | 3.145943057 | 0.001641638 |  |  |
| rs11236797 | 0.470011 | 0.155704 | 0.017 |  |  | 3.164558105 | 0.001206086 |  |  |
| rs140892874 | 0.027461 | 0.409594 | 0.0512 |  |  | 9.53090441 | 9.86489E-05 |  |  |
| rs2193041 | 0.388781 | 0.133703 | 0.0172 |  |  | 3.2017882 | 0.00082876 |  |  |
| rs3850378 | 0.096461 | 0.155099 | 0.0282 |  |  | 5.249443445 | 0.000152167 |  |  |
| rs56062135 | 0.236922 | 0.1509 | 0.0198 |  |  | 3.68577944 | 0.000606072 |  |  |
| rs9934775 | 0.16068 | -0.139595 | 0.0232 |  |  | 4.318691061 | 0.000281809 |  |  |
| rs72798422 | 0.041718 | 0.2776 | 0.0431 |  |  | 8.023085549 | 9.572E-05 |  |  |
| rs2076756 | 0.27064 | 0.187595 | 0.0186 |  |  | 3.462398868 | 0.001158917 |  |  |
| rs11548656 | 0.034026 | -0.292796 | 0.0507 |  |  | 9.437829172 | 6.32692E-05 |  |  |
| rs12446550 | 0.409666 | 0.107796 | 0.0171 |  |  | 3.183173153 | 0.00055468 |  |  |
| rs3091316 | 0.267193 | -0.112497 | 0.0191 |  |  | 3.555474106 | 0.000392041 |  |  |
| rs12936409 | 0.476383 | 0.145701 | 0.0168 |  |  | 3.12732801 | 0.001082875 |  |  |
| rs744166 | 0.409963 | -0.120703 | 0.0172 |  |  | 3.2017882 | 0.000687551 |  |  |
| rs2542147 | 0.837193 | -0.1513 | 0.0227 |  |  | 4.225615823 | 0.000349483 |  |  |
| rs142770866 | 0.0810417 | 0.23 | 0.0337 |  |  | 6.273271067 | 0.000200217 |  |  |
| rs10408351 | 0.238435 | 0.137804 | 0.0221 |  |  | 4.113925537 | 0.000407491 |  |  |
| rs6062496 | 0.578011 | 0.164997 | 0.018 |  |  | 3.350708582 | 0.001182894 |  |  |
| rs1736161 | 0.428732 | -0.123298 | 0.0174 |  |  | 3.239018296 | 0.000709808 |  |  |
| rs8134436 | 0.603989 | -0.144698 | 0.017 |  |  | 3.164558105 | 0.00100015 |  |  |
| rs2836882 | 0.257617 | -0.196295 | 0.0201 |  |  | 3.741624583 | 0.001052763 |  |  |
| rs7285952 | 0.157193 | -0.176002 | 0.0235 |  |  | 4.374536204 | 0.000428906 |  |  |
| rs1003342 | 0.531334 | -0.095001 | 0.0168 |  |  | 3.12732801 | 0.00045959 |  |  |
| rs131657 | 0.201666 | 0.136496 | 0.0212 |  |  | 3.946390107 | 0.000385201 |  |  |
| SD, standard deviation; SNP, single nucleotide polymorphisms. | | | |  |  |  |  |  |  |
