# Supplementary material for: Two-sample Mendelian randomization study reveals no causal relationship between inflammatory bowel disease and urological cancers
Source: Front Genet. 2023 Dec 21;14:1275247. doi: 10.3389/fgene.2023.1275247 (PMC10771298; doi:10.3389/fgene.2023.1275247)
Supplement: Supplementary file 6 [file Table2.DOCX]

| **Table S2. F‐statistics to evaluate the instrument strength on Crohn's disease.** | | | | | | | | | |
| --- | --- | --- | --- | --- | --- | --- | --- | --- | --- |
| SNP | eaf.exposure | beta.exposure | se.exposure | sample（N） | K | SD | R2 | sum(R2) | F |
| rs6588243 | 0.589982 | 0.131704 | 0.0234 | 20883 | 52 | 3.381522657 | 0.000733914 | 0.054779404 | 23.21507318 |
| rs7543234 | 0.238986 | 0.155498 | 0.0267 |  |  | 3.858404057 | 0.000590785 |  |  |
| rs697693 | 0.201415 | 0.172296 | 0.0281 |  |  | 4.060717379 | 0.000579146 |  |  |
| rs3024505 | 0.16156 | 0.177903 | 0.0302 |  |  | 4.364187361 | 0.00045019 |  |  |
| rs6704109 | 0.256123 | 0.202002 | 0.0256 |  |  | 3.699443591 | 0.001136104 |  |  |
| rs11209026 | 0.0563213 | -0.995199 | 0.0639 |  |  | 9.234158025 | 0.001234673 |  |  |
| rs4851586 | 0.759729 | -0.168899 | 0.0261 |  |  | 3.771698348 | 0.0007321 |  |  |
| rs112401990 | 0.373412 | 0.132203 | 0.0237 |  |  | 3.424875512 | 0.000697258 |  |  |
| rs12692254 | 0.543391 | 0.301402 | 0.0232 |  |  | 3.352620754 | 0.004010609 |  |  |
| rs78487399 | 0.100704 | 0.2259 | 0.037 |  |  | 5.346852065 | 0.000323307 |  |  |
| rs1873625 | 0.320264 | 0.180704 | 0.0243 |  |  | 3.511581221 | 0.001152945 |  |  |
| rs13135092 | 0.095419 | 0.221495 | 0.0389 |  |  | 5.621420144 | 0.000268008 |  |  |
| rs6873866 | 0.534873 | -0.168096 | 0.0239 |  |  | 3.453777415 | 0.001178632 |  |  |
| rs12717899 | 0.793845 | 0.159198 | 0.0289 |  |  | 4.176324991 | 0.000475605 |  |  |
| rs147018773 | 0.0966978 | 0.321699 | 0.0375 |  |  | 5.419106822 | 0.000615635 |  |  |
| rs2188962 | 0.439546 | 0.212398 | 0.0228 |  |  | 3.294816948 | 0.002047447 |  |  |
| rs7714401 | 0.336701 | 0.159403 | 0.0244 |  |  | 3.526032172 | 0.00091286 |  |  |
| rs7713270 | 0.623824 | 0.296602 | 0.0241 |  |  | 3.482679318 | 0.003404116 |  |  |
| rs140054334 | 0.038307 | 0.349797 | 0.0628 |  |  | 9.075197558 | 0.000109462 |  |  |
| rs148844907 | 0.00828273 | 0.958001 | 0.1419 |  |  | 20.50590022 | 3.58563E-05 |  |  |
| rs444210 | 0.546838 | 0.163402 | 0.0229 |  |  | 3.309267899 | 0.001208351 |  |  |
| rs114607072 | 0.0402795 | 0.441797 | 0.0629 |  |  | 9.08964851 | 0.000182646 |  |  |
| rs28701841 | 0.116563 | 0.224303 | 0.0373 |  |  | 5.390204919 | 0.000356637 |  |  |
| rs12194825 | 0.18587 | -0.171904 | 0.0298 |  |  | 4.306383555 | 0.000482258 |  |  |
| rs1456896 | 0.697556 | 0.139301 | 0.0251 |  |  | 3.627188833 | 0.000622332 |  |  |
| rs921720 | 0.618841 | 0.162895 | 0.0237 |  |  | 3.424875512 | 0.001067189 |  |  |
| rs4077515 | 0.419834 | 0.215901 | 0.0235 |  |  | 3.395973609 | 0.001968977 |  |  |
| rs3810936 | 0.698408 | 0.207799 | 0.0263 |  |  | 3.800600251 | 0.001259336 |  |  |
| rs1887428 | 0.623303 | -0.168099 | 0.0243 |  |  | 3.511581221 | 0.001076084 |  |  |
| rs1250573 | 0.287447 | -0.170895 | 0.0264 |  |  | 3.815051203 | 0.000821983 |  |  |
| rs2505640 | 0.643588 | -0.145701 | 0.0237 |  |  | 3.424875512 | 0.000830282 |  |  |
| rs10761659 | 0.55269 | 0.212006 | 0.0237 |  |  | 3.424875512 | 0.001894641 |  |  |
| rs10748781 | 0.55088 | -0.219102 | 0.0238 |  |  | 3.439326463 | 0.002008146 |  |  |
| rs11236797 | 0.473264 | 0.181104 | 0.0231 |  |  | 3.338169802 | 0.001467458 |  |  |
| rs11564236 | 0.034419 | 0.519093 | 0.0595 |  |  | 8.598316158 | 0.000242259 |  |  |
| rs1932990 | 0.253841 | 0.152901 | 0.0263 |  |  | 3.800600251 | 0.000613111 |  |  |
| rs4902642 | 0.409159 | -0.129198 | 0.0236 |  |  | 3.41042456 | 0.000693885 |  |  |
| rs56062135 | 0.234271 | 0.193097 | 0.0269 |  |  | 3.88730596 | 0.000885273 |  |  |
| rs147684209 | 0.369415 | 0.154901 | 0.0244 |  |  | 3.526032172 | 0.000899133 |  |  |
| rs72798422 | 0.047701 | 0.590392 | 0.0508 |  |  | 7.341083375 | 0.000587613 |  |  |
| rs2076756 | 0.283672 | 0.399806 | 0.0242 |  |  | 3.497130269 | 0.005311698 |  |  |
| rs744166 | 0.408159 | -0.129299 | 0.0233 |  |  | 3.367071706 | 0.000712443 |  |  |
| rs3091315 | 0.265588 | -0.179501 | 0.0263 |  |  | 3.800600251 | 0.000870177 |  |  |
| rs907092 | 0.471412 | 0.130396 | 0.0228 |  |  | 3.294816948 | 0.000780574 |  |  |
| rs80262450 | 0.112701 | 0.283102 | 0.0353 |  |  | 5.101185889 | 0.000615987 |  |  |
| rs281379 | 0.489275 | 0.139797 | 0.0238 |  |  | 3.439326463 | 0.000825694 |  |  |
| rs2129944 | 0.291299 | -0.1562 | 0.0271 |  |  | 3.916207863 | 0.000656845 |  |  |
| rs8178977 | 0.238841 | 0.1928 | 0.0274 |  |  | 3.959560718 | 0.000862054 |  |  |
| rs1056441 | 0.697697 | 0.167 | 0.0255 |  |  | 3.684992639 | 0.000866361 |  |  |
| rs7276302 | 0.608306 | -0.171598 | 0.0231 |  |  | 3.338169802 | 0.001259234 |  |  |
| rs1297271 | 0.429877 | -0.154901 | 0.0237 |  |  | 3.424875512 | 0.001002678 |  |  |
| rs4820091 | 0.197556 | 0.171702 | 0.0282 |  |  | 4.07516833 | 0.000562852 |  |  |
| rs151314883 | 0.157585 | -0.223994 | 0.0327 |  |  | 4.725461149 | 0.000596562 |  |  |
| SD, standard deviation; SNP, single nucleotide polymorphisms. | | | |  |  |  |  |  |  |
