# Supplementary material for: Two-sample Mendelian randomization study reveals no causal relationship between inflammatory bowel disease and urological cancers
Source: Front Genet. 2023 Dec 21;14:1275247. doi: 10.3389/fgene.2023.1275247 (PMC10771298; doi:10.3389/fgene.2023.1275247)
Supplement: Supplementary file 7 [file Table5.DOCX]

| **Table S5. Casual effect of Crohn's disease on urological cancers in the MR analyses.** | | | | | | | | |
| --- | --- | --- | --- | --- | --- | --- | --- | --- |
| **Cancer site** | **Databases** | **Method** | **β** | **SE** | **Pval** | **OR** | **LCI 95** | **UCI 95** |
| Kidney | UK Biobank | MR Egger | 0.00015 | 0.00021 | 0.48238 | 1.00015 | 0.99974 | 1.00055 |
|  |  | Weighted median | 0.00002 | 0.00014 | 0.90633 | 1.00002 | 0.99974 | 1.00029 |
|  |  | Inverse variance weighted | 0.00007 | 0.00009 | 0.46841 | 1.00007 | 0.99989 | 1.00024 |
|  |  | Simple mode | 0.00005 | 0.00025 | 0.84279 | 1.00005 | 0.99957 | 1.00053 |
|  |  | Weighted mode | 0.00002 | 0.00015 | 0.88321 | 1.00002 | 0.99972 | 1.00033 |
|  | FinnGen | MR Egger | 0.08714 | 0.07300 | 0.23965 | 1.09104 | 0.94559 | 1.25887 |
|  |  | Weighted median | -0.02548 | 0.04528 | 0.57362 | 0.97484 | 0.89206 | 1.06531 |
|  |  | Inverse variance weighted | -0.01790 | 0.02967 | 0.54631 | 0.98226 | 0.92677 | 1.04107 |
|  |  | Simple mode | -0.02515 | 0.09932 | 0.80136 | 0.97516 | 0.80267 | 1.18473 |
|  |  | Weighted mode | 0.08516 | 0.07563 | 0.26671 | 1.08889 | 0.93887 | 1.26289 |
| Bladder | UK Biobank | MR Egger | 0.00019 | 0.00032 | 0.55603 | 1.00019 | 0.99956 | 1.00081 |
|  |  | Weighted median | 0.00003 | 0.00021 | 0.87059 | 1.00003 | 0.99963 | 1.00044 |
|  |  | Inverse variance weighted | -0.00005 | 0.00014 | 0.70414 | 0.99995 | 0.99967 | 1.00022 |
|  |  | Simple mode | 0.00008 | 0.00036 | 0.82572 | 1.00008 | 0.99938 | 1.00078 |
|  |  | Weighted mode | 0.00015 | 0.00025 | 0.55449 | 1.00015 | 0.99966 | 1.00063 |
|  | FinnGen | MR Egger | 0.06135 | 0.07166 | 0.39704 | 1.06327 | 0.92395 | 1.22359 |
|  |  | Weighted median | 0.01053 | 0.03848 | 0.78440 | 1.01058 | 0.93717 | 1.08974 |
|  |  | Inverse variance weighted | -0.00342 | 0.02904 | 0.90622 | 0.99659 | 0.94145 | 1.05495 |
|  |  | Simple mode | 0.00707 | 0.06660 | 0.91599 | 1.00709 | 0.88385 | 1.14752 |
|  |  | Weighted mode | 0.00169 | 0.05199 | 0.97428 | 1.00169 | 0.90465 | 1.10914 |
| Prostate | UK Biobank | MR Egger | -0.00145 | 0.00119 | 0.22938 | 0.99855 | 0.99624 | 1.00088 |
|  |  | Weighted median | -0.00070 | 0.00073 | 0.33291 | 0.99930 | 0.99787 | 1.00072 |
|  |  | Inverse variance weighted | -0.00079 | 0.00052 | 0.12482 | 0.99921 | 0.99820 | 1.00022 |
|  |  | Simple mode | -0.00053 | 0.00144 | 0.71760 | 0.99947 | 0.99665 | 1.00231 |
|  |  | Weighted mode | -0.00072 | 0.00089 | 0.42067 | 0.99928 | 0.99754 | 1.00102 |
|  | FinnGen | MR Egger | -0.02332 | 0.03640 | 0.52544 | 0.97695 | 0.90968 | 1.04920 |
|  |  | Weighted median | -0.00227 | 0.02089 | 0.91353 | 0.99773 | 0.95770 | 1.03944 |
|  |  | Inverse variance weighted | 0.00487 | 0.01472 | 0.74083 | 1.00488 | 0.97631 | 1.03429 |
|  |  | Simple mode | 0.01215 | 0.03839 | 0.75315 | 1.01223 | 0.93886 | 1.09132 |
|  |  | Weighted mode | 0.00047 | 0.02808 | 0.98670 | 1.00047 | 0.94689 | 1.05708 |
|  | PRACTICAL | MR Egger | -0.00523 | 0.01964 | 0.79133 | 0.99478 | 0.95721 | 1.03383 |
|  |  | Weighted median | 0.01158 | 0.01124 | 0.30322 | 1.01164 | 0.98959 | 1.03419 |
|  |  | Inverse variance weighted | 0.00223 | 0.00855 | 0.79465 | 1.00223 | 0.98557 | 1.01917 |
|  |  | Simple mode | 0.02420 | 0.02156 | 0.26846 | 1.02449 | 0.98210 | 1.06872 |
|  |  | Weighted mode | 0.02255 | 0.01681 | 0.18743 | 1.02280 | 0.98965 | 1.05707 |
| PRATICAL, Prostate Cancer Association Group to Investigate Cancer Associated Alterations in the Genome Consortium; SE, standard error; OR, odds ratio; LCI,lower confidence interval; UCI,upper confidence interval. | | | | | | | | |
