# Supplementary material for: Two-sample Mendelian randomization study reveals no causal relationship between inflammatory bowel disease and urological cancers
Source: Front Genet. 2023 Dec 21;14:1275247. doi: 10.3389/fgene.2023.1275247 (PMC10771298; doi:10.3389/fgene.2023.1275247)
Supplement: Supplementary file 8 [file Table3.DOCX]

| **Table S3. F‐statistics to evaluate the instrument strength on ulcerative colitis.** | | | | | | | | | |
| --- | --- | --- | --- | --- | --- | --- | --- | --- | --- |
| SNP | eaf.exposure | beta.exposure | se.exposure | sample（N） | K | SD | R2 | sum(R2) | F |
| rs11209026 | 0.0589321 | -0.561698 | 0.0517 | 27432 | 39 | 8.562868589 | 0.000477277 | 0.034891164 | 25.39208148 |
| rs7523335 | 0.176698 | -0.170397 | 0.0285 |  |  | 4.72034342 | 0.000379138 |  |  |
| rs3024493 | 0.168128 | 0.236297 | 0.0276 |  |  | 4.571279943 | 0.000747424 |  |  |
| rs10917545 | 0.882174 | -0.185101 | 0.0335 |  |  | 5.548473844 | 0.000231364 |  |  |
| rs1886731 | 0.480618 | -0.140501 | 0.0221 |  |  | 3.660336476 | 0.000735586 |  |  |
| rs10737481 | 0.556463 | 0.250104 | 0.0216 |  |  | 3.577523434 | 0.002412528 |  |  |
| rs1801274 | 0.483094 | -0.182896 | 0.0217 |  |  | 3.594086042 | 0.001293316 |  |  |
| rs35730213 | 0.266634 | -0.167 | 0.0245 |  |  | 4.05783908 | 0.000662384 |  |  |
| rs10182512 | 0.34989 | 0.1608 | 0.0223 |  |  | 3.693461693 | 0.00086229 |  |  |
| rs12612675 | 0.402826 | 0.122902 | 0.0219 |  |  | 3.627211259 | 0.000552358 |  |  |
| rs4676410 | 0.197128 | 0.207802 | 0.0284 |  |  | 4.703780811 | 0.000617774 |  |  |
| rs9823546 | 0.310414 | 0.176899 | 0.0223 |  |  | 3.693461693 | 0.000982072 |  |  |
| rs114152040 | 0.0317179 | 0.339603 | 0.0623 |  |  | 10.31850509 | 6.65343E-05 |  |  |
| rs254559 | 0.404112 | 0.124304 | 0.0215 |  |  | 3.560960825 | 0.000586857 |  |  |
| rs56167332 | 0.34162 | 0.151596 | 0.0231 |  |  | 3.825962561 | 0.000706226 |  |  |
| rs7752873 | 0.13681 | 0.182297 | 0.0303 |  |  | 5.018470373 | 0.000311652 |  |  |
| rs9272514 | 0.297965 | -0.401598 | 0.0267 |  |  | 4.422216467 | 0.003450302 |  |  |
| rs28383456 | 0.334505 | -0.337307 | 0.0256 |  |  | 4.240027773 | 0.002817674 |  |  |
| rs113473719 | 0.691824 | -0.259499 | 0.0271 |  |  | 4.488466901 | 0.001425279 |  |  |
| rs6933404 | 0.21562 | 0.166799 | 0.0252 |  |  | 4.17377734 | 0.000540224 |  |  |
| rs148844907 | 0.010151 | 1.3413 | 0.1089 |  |  | 18.03668065 | 0.000111134 |  |  |
| rs10272963 | 0.426078 | -0.171904 | 0.0216 |  |  | 3.577523434 | 0.001129221 |  |  |
| rs798502 | 0.283142 | -0.136496 | 0.0239 |  |  | 3.958463429 | 0.000482674 |  |  |
| rs989960 | 0.424904 | -0.129096 | 0.0215 |  |  | 3.560960825 | 0.000642321 |  |  |
| rs1887428 | 0.622856 | -0.176701 | 0.0224 |  |  | 3.710024302 | 0.001065735 |  |  |
| rs4574921 | 0.740842 | 0.150599 | 0.0256 |  |  | 4.240027773 | 0.000484425 |  |  |
| rs3829111 | 0.416668 | 0.156303 | 0.0214 |  |  | 3.544398217 | 0.000945335 |  |  |
| rs7911680 | 0.490094 | -0.1718 | 0.0213 |  |  | 3.527835608 | 0.001185302 |  |  |
| rs483905 | 0.294334 | 0.128903 | 0.0228 |  |  | 3.776274736 | 0.000484026 |  |  |
| rs484356 | 0.32765 | -0.134199 | 0.0228 |  |  | 3.776274736 | 0.000556425 |  |  |
| rs2212434 | 0.459604 | 0.141899 | 0.0213 |  |  | 3.527835608 | 0.000803652 |  |  |
| rs12817473 | 0.382176 | 0.190701 | 0.0217 |  |  | 3.594086042 | 0.001329496 |  |  |
| rs1359946 | 0.196096 | 0.158302 | 0.0269 |  |  | 4.455341684 | 0.000398028 |  |  |
| rs9891174 | 0.470398 | 0.145199 | 0.0212 |  |  | 3.511273 | 0.000852007 |  |  |
| rs6017342 | 0.537652 | 0.191306 | 0.024 |  |  | 3.975026038 | 0.001151536 |  |  |
| rs6062496 | 0.572636 | 0.158498 | 0.0224 |  |  | 3.710024302 | 0.000893307 |  |  |
| rs7282490 | 0.603618 | -0.139699 | 0.0214 |  |  | 3.544398217 | 0.000743375 |  |  |
| rs9977672 | 0.251316 | -0.245006 | 0.0261 |  |  | 4.322840816 | 0.001208826 |  |  |
| rs137845 | 0.514874 | 0.118198 | 0.0212 |  |  | 3.511273 | 0.000566078 |  |  |
| SD, standard deviation; SNP, single nucleotide polymorphisms. | | | |  |  |  |  |  |  |
