# Supplementary material for: Two-sample Mendelian randomization study reveals no causal relationship between inflammatory bowel disease and urological cancers
Source: Front Genet. 2023 Dec 21;14:1275247. doi: 10.3389/fgene.2023.1275247 (PMC10771298; doi:10.3389/fgene.2023.1275247)
Supplement: Supplementary file 9 [file Table4.DOCX]

| **Table S4. Casual effect of inflammatory bowel disease on urological cancers in the MR analyses.** | | | | | | | | |
| --- | --- | --- | --- | --- | --- | --- | --- | --- |
| **Cancer site** | **Databases** | **Method** | **β** | **SE** | **Pval** | **OR** | **LCI 95** | **UCI 95** |
| Kidney | UK Biobank | MR-Egger | 0.000120 | 0.000317 | 0.705667 | 1.000120 | 0.999500 | 1.000741 |
|  |  | Weighted median | -0.000001 | 0.000178 | 0.994584 | 0.999999 | 0.999650 | 1.000348 |
|  |  | Inverse variance weighted | 0.000002 | 0.000114 | 0.986314 | 1.000002 | 0.999778 | 1.000226 |
|  |  | Simple mode | 0.000006 | 0.000347 | 0.985297 | 1.000006 | 0.999327 | 1.000686 |
|  |  | Weighted mode | 0.000044 | 0.000253 | 0.862219 | 1.000044 | 0.999548 | 1.000540 |
|  | FinnGen | MR-Egger | 0.199512278 | 0.103658 | 0.060461 | 1.220807 | 0.99635 | 1.49583 |
|  |  | Weighted median | -0.020949758 | 0.053504 | 0.695385 | 0.979268 | 0.881776 | 1.087539 |
|  |  | Inverse variance weighted | -0.01352385 | 0.036922 | 0.714155 | 0.986567 | 0.917694 | 1.060609 |
|  |  | Simple mode | -0.131688722 | 0.131011 | 0.319962 | 0.876614 | 0.678093 | 1.133255 |
|  |  | Weighted mode | -0.128200728 | 0.106905 | 0.236462 | 0.879677 | 0.713384 | 1.084733 |
| Bladder | UK Biobank | MR-Egger | -9.25527E-05 | 0.000518 | 0.858816 | 0.999907 | 0.998893 | 1.000923 |
|  |  | Weighted median | 0.000146893 | 0.000248 | 0.553071 | 1.000147 | 0.999662 | 1.000632 |
|  |  | Inverse variance weighted | -0.000131186 | 0.000187 | 0.48219 | 0.999869 | 0.999503 | 1.000235 |
|  |  | Simple mode | 0.000194885 | 0.000512 | 0.704863 | 1.000195 | 0.999192 | 1.001199 |
|  |  | Weighted mode | 0.000194885 | 0.000384 | 0.613446 | 1.000195 | 0.999443 | 1.000947 |
|  | FinnGen | MR-Egger | 0.007306276 | 0.09948 | 0.941771 | 1.007333 | 0.828884 | 1.2242 |
|  |  | Weighted median | -0.010473103 | 0.046834 | 0.823053 | 0.989582 | 0.902787 | 1.08472 |
|  |  | Inverse variance weighted | 0.007862028 | 0.033976 | 0.817003 | 1.007893 | 0.942961 | 1.077296 |
|  |  | Simple mode | 0.008147944 | 0.090118 | 0.928342 | 1.008181 | 0.844945 | 1.202953 |
|  |  | Weighted mode | 0.004171792 | 0.069725 | 0.952543 | 1.004181 | 0.875912 | 1.151232 |
| Prostate | UK Biobank | MR-Egger | -0.001788884 | 0.001552 | 0.254283 | 0.998213 | 0.995181 | 1.001253 |
|  |  | Weighted median | -0.000986247 | 0.000898 | 0.271939 | 0.999014 | 0.997258 | 1.000774 |
|  |  | Inverse variance weighted | -0.000733756 | 0.000563 | 0.192159 | 0.999267 | 0.998165 | 1.000369 |
|  |  | Simple mode | -0.001965311 | 0.001749 | 0.266222 | 0.998037 | 0.994621 | 1.001464 |
|  |  | Weighted mode | -0.00156598 | 0.001312 | 0.238044 | 0.998435 | 0.995871 | 1.001007 |
|  | FinnGen | MR-Egger | -0.065072381 | 0.052759 | 0.223693 | 0.937 | 0.844949 | 1.039079 |
|  |  | Weighted median | 0.012629029 | 0.025578 | 0.621492 | 1.012709 | 0.96319 | 1.064774 |
|  |  | Inverse variance weighted | 0.01376211 | 0.018536 | 0.457819 | 1.013857 | 0.977684 | 1.051369 |
|  |  | Simple mode | 0.052865909 | 0.048199 | 0.278303 | 1.054288 | 0.95925 | 1.158743 |
|  |  | Weighted mode | 0.030870392 | 0.037512 | 0.414694 | 1.031352 | 0.958244 | 1.110038 |
|  | PRACTICAL | MR-Egger | -0.044288148 | 0.030192 | 0.148792 | 0.956678 | 0.901709 | 1.014999 |
|  |  | Weighted median | -0.011242799 | 0.01358 | 0.407723 | 0.98882 | 0.962849 | 1.015492 |
|  |  | Inverse variance weighted | 0.004862318 | 0.011302 | 0.667027 | 1.004874 | 0.98286 | 1.027382 |
|  |  | Simple mode | -0.018586602 | 0.035094 | 0.598721 | 0.981585 | 0.916337 | 1.05148 |
|  |  | Weighted mode | -0.028762935 | 0.024729 | 0.250301 | 0.971647 | 0.925675 | 1.019901 |
| PRATICAL, Prostate Cancer Association Group to Investigate Cancer Associated Alterations in the Genome Consortium; SE, standard error; OR, odds ratio; LCI,lower confidence interval; UCI,upper confidence interval. | | | | | | | | |
