# Supplementary material for: Two-sample Mendelian randomization study reveals no causal relationship between inflammatory bowel disease and urological cancers
Source: Front Genet. 2023 Dec 21;14:1275247. doi: 10.3389/fgene.2023.1275247 (PMC10771298; doi:10.3389/fgene.2023.1275247)
Supplement: Supplementary file 10 [file Table6.DOCX]

| **Table S6. Casual effect of ulcerative colitis on urological cancers in the MR analyses.** | | | | | | | | |
| --- | --- | --- | --- | --- | --- | --- | --- | --- |
| **Cancer site** | **Databases** | **Method** | **β** | **SE** | **Pval** | **OR** | **LCI 95** | **UCI 95** |
| Kidney | UK Biobank | MR Egger | 0.00048 | 0.00033 | 0.16354 | 1.00048 | 0.99982 | 1.00113 |
|  |  | Weighted median | 0.00014 | 0.00017 | 0.41450 | 1.00014 | 0.99980 | 1.00048 |
|  |  | Inverse variance weighted | 0.00012 | 0.00012 | 0.34295 | 1.00012 | 0.99988 | 1.00035 |
|  |  | Simple mode | 0.00018 | 0.00027 | 0.51194 | 1.00018 | 0.99965 | 1.00070 |
|  |  | Weighted mode | 0.00021 | 0.00020 | 0.30565 | 1.00021 | 0.99981 | 1.00061 |
|  | FinnGen | MR Egger | 0.20515 | 0.15580 | 0.19899 | 1.22771 | 0.90464 | 1.66616 |
|  |  | Weighted median | -0.05054 | 0.05966 | 0.39694 | 0.95072 | 0.84580 | 1.06865 |
|  |  | Inverse variance weighted | 0.01735 | 0.04492 | 0.69930 | 1.01750 | 0.93174 | 1.11116 |
|  |  | Simple mode | -0.06500 | 0.13542 | 0.63499 | 0.93707 | 0.71862 | 1.22193 |
|  |  | Weighted mode | -0.07494 | 0.11111 | 0.50557 | 0.92780 | 0.74623 | 1.15355 |
| Bladder | UK Biobank | MR Egger | 0.00038 | 0.00058 | 0.52252 | 1.00038 | 0.99923 | 1.00152 |
|  |  | Weighted median | 0.00023 | 0.00026 | 0.36763 | 1.00023 | 0.99973 | 1.00074 |
|  |  | Inverse variance weighted | -0.00012 | 0.00021 | 0.55646 | 0.99988 | 0.99946 | 1.00029 |
|  |  | Simple mode | 0.00033 | 0.00047 | 0.48586 | 1.00033 | 0.99941 | 1.00126 |
|  |  | Weighted mode | 0.00038 | 0.00040 | 0.34243 | 1.00038 | 0.99961 | 1.00116 |
|  | FinnGen | MR Egger | 0.02562 | 0.12440 | 0.83836 | 1.02595 | 0.80397 | 1.30924 |
|  |  | Weighted median | 0.02037 | 0.04816 | 0.67236 | 1.02058 | 0.92865 | 1.12161 |
|  |  | Inverse variance weighted | 0.00186 | 0.03542 | 0.95816 | 1.00186 | 0.93467 | 1.07388 |
|  |  | Simple mode | -0.01597 | 0.08528 | 0.85281 | 0.98416 | 0.83266 | 1.16321 |
|  |  | Weighted mode | 0.00942 | 0.07296 | 0.89824 | 1.00946 | 0.87495 | 1.16465 |
| Prostate | UK Biobank | MR Egger | -0.00256 | 0.00211 | 0.23362 | 0.99744 | 0.99333 | 1.00157 |
|  |  | Weighted median | -0.00139 | 0.00090 | 0.12249 | 0.99862 | 0.99686 | 1.00037 |
|  |  | Inverse variance weighted | -0.00011 | 0.00077 | 0.88215 | 0.99989 | 0.99838 | 1.00140 |
|  |  | Simple mode | -0.00203 | 0.00158 | 0.21043 | 0.99797 | 0.99488 | 1.00108 |
|  |  | Weighted mode | -0.00187 | 0.00100 | 0.07032 | 0.99813 | 0.99618 | 1.00008 |
|  | FinnGen | MR Egger | 0.05288 | 0.08407 | 0.53486 | 1.05430 | 0.89413 | 1.24316 |
|  |  | Weighted median | 0.04239 | 0.02648 | 0.10938 | 1.04330 | 0.99054 | 1.09887 |
|  |  | Inverse variance weighted | 0.01769 | 0.02390 | 0.45926 | 1.01785 | 0.97126 | 1.06667 |
|  |  | Simple mode | 0.08047 | 0.04890 | 0.11146 | 1.08379 | 0.98474 | 1.19281 |
|  |  | Weighted mode | 0.08579 | 0.04446 | 0.06423 | 1.08958 | 0.99865 | 1.18879 |
|  | PRACTICAL | MR Egger | -0.03098 | 0.04134 | 0.46011 | 0.96949 | 0.89403 | 1.05133 |
|  |  | Weighted median | -0.02530 | 0.01560 | 0.10497 | 0.97502 | 0.94566 | 1.00530 |
|  |  | Inverse variance weighted | -0.01351 | 0.01480 | 0.36109 | 0.98658 | 0.95838 | 1.01561 |
|  |  | Simple mode | -0.01436 | 0.03741 | 0.70395 | 0.98574 | 0.91604 | 1.06074 |
|  |  | Weighted mode | -0.04477 | 0.02118 | 0.04354 | 0.95622 | 0.91734 | 0.99674 |
| PRATICAL, Prostate Cancer Association Group to Investigate Cancer Associated Alterations in the Genome Consortium; SE, standard error; OR, odds ratio; LCI,lower confidence interval; UCI,upper confidence interval. | | | | | | | | |
